# Administration of Tonsil-Derived Mesenchymal Stem Cells Improves Glucose Tolerance in High Fat Diet-Induced Diabetic Mice via Insulin-Like Growth Factor-Binding Protein 5-Mediated Endoplasmic Reticulum Stress Modulation

**DOI:** 10.3390/cells8040368

**Published:** 2019-04-23

**Authors:** Younghay Lee, Sun-Hye Shin, Kyung-Ah Cho, Yu-Hee Kim, So-Youn Woo, Han Su Kim, Sung-Chul Jung, Inho Jo, Hee-Sook Jun, Woo-Jae Park, Joo-Won Park, Kyung-Ha Ryu

**Affiliations:** 1 Department of Biochemistry, College of Medicine, Ewha Womans University, Seoul 07804, Korea; younghay33@naver.com (Y.L.); s1sunhye@naver.com (S.-H.S.); jungsc@ewha.ac.kr (S.-C.J.); 2 Department of Microbiology, College of Medicine, Ewha Womans University, Seoul 07804, Korea; kyungahcho@ewha.ac.kr (K.-A.C.); kimyuhee@ewha.ac.kr (Y.-H.K.); soyounwoo@ewha.ac.kr (S.-Y.W.); 3 Department of Otolaryngology, College of Medicine, Ewha Womans University, Seoul 07985, Korea; sevent@ewha.ac.kr; 4 Department of Molecular Medicine, College of Medicine, Ewha Womans University, Seoul 07804, Korea; inhojo@ewha.ac.kr; 5 College of Pharmacy and Gachon Institute of Pharmaceutical Science, Gachon University, Incheon 21999, Korea; hsjun@gachon.ac.kr; 6 Department of Biochemistry, College of Medicine, Gachon University, Incheon 21999, Korea; ooze@gachon.ac.kr; 7 Department of Pediatrics, College of Medicine, Ewha Womans University, Seoul 07804, Korea

**Keywords:** type 2 diabetes mellitus, tonsil, mesenchymal stem cell, pancreas, insulin-like growth factor-binding protein 5

## Abstract

Type 2 diabetes mellitus (T2DM) is a prevalent chronic metabolic disorder accompanied by high blood glucose, insulin resistance, and relative insulin deficiency. Endoplasmic reticulum (ER) stress induced by high glucose and free fatty acids has been suggested as one of the main causes of β-cell dysfunction and death in T2DM. Stem cell-derived insulin-secreting cells were recently suggested as a novel therapy for diabetes. In the present study, we demonstrate the therapeutic potential of tonsil-derived mesenchymal stem cells (TMSCs) to treat high-fat diet (HFD)-induced T2DM. To explore whether TMSC administration can alleviate T2DM, TMSCs were intraperitoneally injected in HFD-induced T2DM mice once every 2 weeks. TMSC injection markedly improved glucose tolerance and glucose-stimulated insulin secretion and prevented HFD-induced pancreatic β-cell hypertrophy and cell death. In addition, TMSC injection relieved the ER-stress response and preserved gene expression related to glucose sensing and insulin secretion. Moreover, administration of TMSC-derived conditioned medium induced similar therapeutic outcomes, suggesting paracrine effects. Finally, proteomic analysis revealed high secretion of insulin-like growth factor-binding protein 5 by TMSCs, and its expression was critical for the protective effects of TMSCs against HFD-induced glucose intolerance and ER-stress response in pancreatic islets. TMSC administration can alleviate HFD-induced-T2DM via preserving pancreatic islets and their function. These results provide novel evidence of TMSCs as an ER-stress modulator that may be a novel, alternative cell therapy for T2DM.

## 1. Introduction

Type 2 diabetes mellitus (T2DM) is a heterogeneous metabolic disease characterized by hyperglycaemia due to insulin resistance in adipose tissue, muscle, and liver and/or impaired insulin secretion by pancreatic β-cells [1]. While insulin resistance and obesity are mainly associated with T2DM, diabetic symptoms only develop in insulin-resistant individuals following the onset of β-cell dysfunction [1]. Furthermore, the natural history of T2DM entails progressive deterioration of β-cell function, associated with loss of β-cell mass [2,3]. The molecular mechanisms underlying β-cell failure in T2DM reportedly result from endoplasmic reticulum (ER)-stress responses induced by glucolipotoxicity [4] and autophagy dysfunction related to amyloid accumulation [5]. While currently available T2DM treatments, including oral anti-diabetic drugs and insulin subcutaneous injection, can alleviate hyperglycaemia or temporarily improve insulin sensitivity in target tissues, these can neither reverse insulin resistance nor the progressive β-cell dysfunction; that is, none of these therapies modulate the course of the disease [6].

Stem cell therapy has recently emerged as one of the most potent therapeutic candidates for numerous human diseases. Mesenchymal stem cells (MSCs) are non-haematopoietic, multi-potent stem cells with the ability to differentiate into mesodermal lineage such as osteocytes, adipocytes, and chondrocytes [7]. They can be easily isolated from various tissues including adipose tissue, amniotic fluid, umbilical cord, and Wharton’s jelly [7]. The immunosuppressive capacity of MSCs enables allogenic transplantation without immunosuppressive drugs, and this improves the clinical utility of MSC-based therapies. We previously reported the feasibility and therapeutic potential of tonsil-derived MSCs (TMSCs) in various disease murine models such as immune-mediated hepatitis [8], liver fibrosis [9], osteoporosis [10,11], and colitis [12]. Palatine tonsil has been considered as an attractive source of MSCs, since TMSCs exhibit several advantages compared with MSCs from other sources. TMSCs can be noninvasively isolated from abandoned tissues of tonsillectomy, a surgical procedure mostly performed on young children to treat recurrent throat infections and sleep-disordered breathing [13], and have a shorter doubling time and faster division compared with adipose tissue-derived MSCs (AMSCs) and bone marrow-derived MSCs (BM-MSCs), possibly due to the young donor age [14,15]. In addition, the stemness characteristics of TMSCs are maintained up to passage 15 [16], and mixed chimerism was successfully formed when TMSCs from three independent donors are mixed-cultured, indicating the feasibility of TMSC banking [17]. In addition, RNA sequencing analyses of BM-MSCs, AMSCs, and TMSCs revealed higher expression levels of genes related with positive cell regulation, cell division, and cell adhesion, which may indicate TMSC transplantation into injured tissue as a promising therapy [15]. Moreover, TMSCs can be differentiated into various cell types including parathyroid cells [18], hepatocytes [9], myocytes [19], tenocytes [20], and neuronal cells [21]. TMSCs can also be efficiently differentiated into functional insulin-producing cells, and administration of these cells into streptozotocin (STZ)-induced type 1 diabetic mice improved glucose intolerance [14]. While the efficacy of MSC-differentiated insulin-producing cells in type 1 diabetes is relatively well established, the therapeutic effects of undifferentiated MSCs in T2DM are only now being evaluated. BM-MSC infusion 7 days after STZ injection not only promoted β-cell function but also ameliorated insulin resistance, whereas infusion at 21 days after STZ injection merely ameliorated insulin resistance in a rat model of diabetes induced by a high-fat diet (HFD)/STZ administration [22]. Similarly, a single intravenous infusion of AMSCs could improve glucose tolerance with better-preserved pancreatic β-cell mass in HFD-induced T2DM mice [23]. 

Since TMSCs exhibit unique properties compared with MSCs from other sources, we examined the therapeutic efficacy and mechanism of TMSCs in HFD-induced T2DM in the present study. Following tail vein intravenous transfusion in mice, the bulk of MSCs are immediately trapped in the lung microvasculature, and only a small proportion redistributes to sites of injury or damage [24], and intravenous MSC infusion can cause fatal pulmonary embolism. Therefore, we performed intraperitoneal injection for TMSC administration. We also analysed the secretome of human TMSCs and demonstrated that insulin-like growth factor-binding protein 5 (IGFBP5) is critical for the therapeutic effect of TMSCs in a HFD-induced diabetes mouse model.

## 2. Materials and Methods

### 2.1. Animal Experiments 

All animal experiment procedures were approved by the Animal Care and Use Committee of the Ewha Womans University School of Medicine (ESM17-0383), and performed in accordance with the Animal Care Guidelines of the Ewha Womans University School of Medicine (Seoul, South Korea) and the National Institutes of Health (NIH; Bethesda, MD, USA) Guidelines for Animal care. BALB/c mice (male, 8 weeks old) were purchased from OrientBio (Seongnam, South Korea) and maintained under specific pathogen-free conditions on a 12-h light/dark cycle. A previous study reported that diabetes-like symptoms can be induced by feeding a HFD for a minimum of 6 weeks [25]. Mice were therefore fed a HFD (60 kcal% fat; Research Diets, New Brunswick, NJ, Canada) for 6 weeks to induce T2DM-like conditions, and healthy control mice were fed a normal chow diet (10 kcal% fat; Research Diets). Intraperitoneal injection of TMSCs or IGFBP5-knockdown TMSCs (2 × 10^6^ cells/mouse) were administered once every 2 weeks, starting 6 weeks after HFD initiation, whereas conditioned medium (CM) was injected via tail vein once a week. Total duration of TMSC injection or CM infusion was 10 weeks, and data was obtained after 10 weeks of TMSC or CM injection. Each group consisted of 10 mice. Fasting blood glucose levels were measured using an automatic glucometer (ACCU-CHECK PERFORMA, Roche Diagnostics, Basel, Switzerland) after 8 h of fasting, and body weight was measured weekly. 

### 2.2. Glucose and Insulin Tolerance Testing

Glucose and insulin tolerance testing was performed as previously described [26] with some modifications. Briefly, either glucose (2.0 g/kg) or insulin (0.75 IU/kg) was injected intraperitoneally after 8 h of fasting. Blood was obtained though the tail vein, and glucose levels were measured using a glucometer (Roche Diagnostics). 

### 2.3. Glucose-Stimulated Insulin Secretion

Pancreatic islets were obtained as described previously [27] with some modifications. GSIS assays were performed with the obtained pancreatic islets as previously described [14]. Briefly, pancreatic islets were washed carefully with phosphate-buffered saline and stabilized in 5 mM Ca^2+^-containing HEPES-added Krebs-Ringer bicarbonate buffer (HKRB) (129 mM NaCl, 1.2 mM MgSO_4_, 1.2 mM KH_2_PO_4_, 4.7 mM KCl, 5 mM NaHCO_3_, 2.5 mM CaCl_2_, 10 mM HEPES (pH 7.4) containing 0.05% bovine serum albumin) with 2.2 mM glucose for 2 h. Islets were washed with HKRB once, and then incubated for 1 h in HKRB with 2.2 or 22 mM of glucose. The supernatant was collected for measuring secreted insulin using an ultrasensitive insulin enzyme-linked immunosorbent assay (ELISA) kit (Crystal Chem, Downers Grove, IL, USA).

### 2.4. Histology and Immunofluorescence

Pancreas, adipose, and liver tissues were fixed in 10% formaldehyde and embedded in paraffin. Sections of adipose and liver tissues (4 μm) were mounted on slides and stained with haematoxylin and eosin. For immunofluorescence, 4-μm sections of pancreatic tissues were incubated overnight at 4 °C with an anti-insulin antibody (Sigma-Aldrich, Saint Louis, MO, USA), and followed by incubation with Cy2-conjugated secondary antibody (Jackson Immuno Research, West Grove, PA, USA). Finally, tissues were mounted with 4′,6-diamidino-2-phenylindole for nuclear staining. Islet density and size were calculated using ImageJ software (NIH).

### 2.5. Cell Culture 

Previously isolated and characterized human TMSCs [8,14,17] were cultured in Dulbecco’s modified Eagle’s medium supplemented with 10% fetal bovine serum (Corning, Corning, NY, USA) and 1% penicillin/streptomycin. The protocol, including informed consent templates, was approved by the Ewha Womans University Medical Center institutional review board (ECT-11-53-02). Briefly, tonsils extracted during tonsillectomies performed in Ewha Womans University Mok-Dong Hospital were digested using collagenase type I (Invitrogen, Carlsbad, CA, USA) and DNase (Sigma-Aldrich). Then, cells from tonsils were collected by Ficoll-Paque (GE Healthcare, Little Chalfont, UK) density gradient centrifugation, and cultured for 48 h, and only adherent cells were replenished with fresh culture medium. For adipogenic differentiation, cells were cultured in adipogenic medium (Invitrogen) for 3 weeks, and stained with Oil Red O (Sigma-Aldrich). For osteogenic differentiation, cells were cultured in osteogenic culture medium (Invitrogen) for 3 weeks, and then stained with 2% Alizarin Red S solution (pH 4.2). For chondrogenic differentiation, cells were pelleted, and cultured in chondrogenic medium (Invitrogen) for 3 weeks. Then, cell pellets were fixed in 4% paraformaldehyde solution and embedded in paraffin. Sections were then stained with an anti-human collagen type II (Abcam, Cambridge, UK) antibody, and followed by incubation with a peroxidase-conjugated secondary antibody (DakoCytomation, Glostrup, Denmark). Finally, sections were counterstained with hematoxylin as described previously [17]. siRNA for IGFBP5 (Invitrogen) was transfected using Metafectene reagents (Biontex Laboratories GmbH, Munich, Germany). Human AMSCs were purchased from Invitrogen and cultured as described previously [14]. Cells were used at passages 5–8.

### 2.6. CM Preparation

CM was prepared as described previously [28]. AMSCs and TMSCs were grown to 85% confluence in 100-mm tissue culture plates, and then cells were washed twice with PBS and cultured in serum-free Dulbecco’s modified Eagle’s medium for 48 h to generate CM. Collected medium was concentrated 50-fold by centrifugal filtration (2K cut-off; Vivaspin, Sartorius, Göttingen, Germany).

### 2.7. MTT 

MTT assays were performed as described previously [29]. Briefly, cells were incubated in medium with 0.5 μg/mL MTT solution for 4 h. Then, the cell medium was removed, and an equal volume of dimethyl sulphoxide was added to dissolve the formazan crystals. The solutions were transferred in 96-well plates to measure absorbance at 560 nm. 

### 2.8. Real-Time Polymerase Chain Reaction (PCR) 

Total mRNA was extracted from adipose tissue, liver, and pancreas using either NucleoSpin RNA (Macherey–Nagel GmbH, Düren, Germany) or Direct-zol RNA MiniPrep (Zymo Research, Irvine, CA, USA). Complementary DNA (cDNA) was synthesized using ReverTraAce qPCR RT master mix with gDNA remover (Toyobo, Japan). Relative gene expressions were determined using the SYBR system (Applied Biosystems, Foster City, CA, USA). Primers used are listed in Supplementary Table S1. 

### 2.9. Western Blot Analyses

Cells were lysed and homogenized at 4 °C in RIPA buffer (50 mM Tris-Cl (pH 7.5), 150 mM NaCl, 1% Nonidet P-40, 0.5% sodium deoxycholate, 0.1% sodium dodecyl sulphate (SDS)) containing 50 mM NaF, 2 mM Na_3_VO_4_, protease inhibitors (Sigma-Aldrich). Protein (50 µg) from cell lysates was loaded in each lane. To compare secreted IGFBP5 levels from TMSCs and AMSCs, equal amounts (20 μL per lane) of CM were subjected to western blot analysis as described previously [28]. Proteins were separated by SDS-polyacrylamide gel electrophoresis, and then transferred to nitrocellulose membranes. The membranes were incubated with the antibodies against IGFBP5 (Santa Cruz Biotechnology, Dallas, TX, USA) and α-tubulin (Sigma-Aldrich). After washing, the membranes were incubated with the corresponding secondary peroxidase-conjugated secondary antibodies (Jackson ImmunoResearch). For chemiluminescence, SuperSignal West Pico chemiluminescent substrate (Thermo Fisher Scientific, Waltham, MA, USA) was used and bands were detected by a Bio-Imaging Analyzer LAS-4000 (Fuji, Tokyo, Japan). 

### 2.10. ELISA

Insulin levels were measured with an ultrasensitive insulin ELISA immunoassay kit (Crystal Chem) according to the manufacturer’s instructions. Tumor necrosis factor-α (TNF-α) levels in serum were measured using an ELISA kit (BioLegend, San Diego, CA, USA) according to the manufacturer׳s protocols.

### 2.11. Cholesterol and Triglyceride Measurements

Cholesterol and triglyceride levels in serum were measured using a Reflotron Plus machine (Roche Diagnostics, Germany).

### 2.12. Fluorescence-Activated Cell Sorting

Immunophenotypes of IGFBP5-knockdown TMSCs were analysed as described previously [14] using a FACS Calibur Cytofluorimeter (BD Biosciences, Franklin Lakes, NJ, USA). 

### 2.13. Microarray Analysis

The microarray process was executed using the GeneChip Whole Transcript PLUS reagent kit (Affymetrix, Santa Clara, CA, USA) according to the manufacturer’s protocol. Complementary DNA was synthesized using the GeneChip Whole Transcript Amplification kit (Affymetrix) as described by the manufacturer. The sense cDNA was then fragmented and biotin labelled with terminal deoxynucleotidyl transferase using the GeneChip whole transcript terminal labelling kit (Affymetrix). Approximately 5.5 μg of labelled DNA target was hybridized to the Affymetrix GeneChip Human 2.0 ST Array at 45 °C for 16 h. Hybridized arrays were washed and stained on a GeneChip Fluidics Station 450 and scanned on a GCS3000 Scanner (Affymetrix). Signal values were computed using Affymetrix GeneChip Command Console software.

### 2.14. Proteomics

Nano liquid chromatography–mass spectrometry (MS)/MS analysis was performed with a nano high-performance liquid chromatography system (Agilent, Wilmington, DE, USA). The nano chip column (Agilent, 150 mm × 0.075 mm) was used for peptide separation. The mobile phase A for liquid chromatography separation was 0.1% formic acid in deionized water, and the mobile phase B was 0.1% formic acid in acetonitrile. The chromatography gradient was designed for a linear increase from 3% B to 45% B in 70 min, 45% B to 95% B in 1 min, 95% B in 9 min, and 3% B in 10 min. The flow rate was maintained at 300 nL/min. Product ion spectra were collected in the information-dependent acquisition mode and were analysed by Agilent 6530 Accurate-Mass Q-TOF using continuous cycles of one full scan time of flight (TOF) MS from 300–2000 *m*/*z* (1.0 s) plus three product ion scans from 150–2000 *m*/*z* (1.5 s each). Precursor *m*/*z* values were selected starting with the most intense ion, using a selection quadrupole resolution of 3 Da. The rolling collision energy feature was used, which determines collision energy based on the precursor value and charge state. The dynamic exclusion time for precursor ion *m*/*z* values was 60 s. The mascot algorithm (Matrixscience, Boston, MA, USA) was used to identify peptide sequences present in a protein sequence database.

### 2.15. Statistical Analysis

The results were expressed as mean ± standard error of the mean (S.E.M.) values. Statistical significance was calculated using the Student’s *t* test, one-way analysis of variance, or repeated-measurements two-way analysis of variance with a post hoc Student’s *t* test. GraphPad PRISM 6 statistical software (GraphPad Software) was used for the analysis. A *p* value < 0.05 was considered significant.

## 3. Results

### 3.1. Intraperitoneal Administration of TMSCs Alleviated HFD-Induced Glucose Intolerance

**Figure 1:**
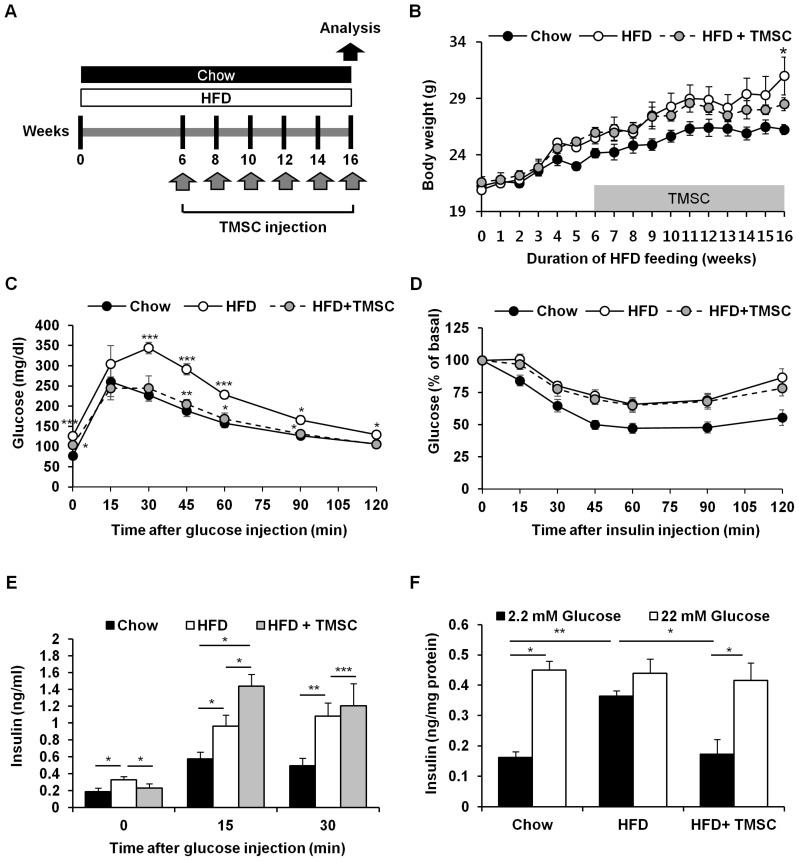
Intraperitoneal tonsil-derived mesenchymal stem cell (TMSC) injection protected mice from HFD-induced glucose intolerance. (**A**) Mice were fed a high fat diet (HFD) for 6 weeks, and then TMSCs were injected intraperitoneally once every 2 weeks into HFD-induced diabetic mice while a normal or HFD diet was maintained. (**B**) Body weights were measured during chow or HFD feeding (*n* = 10). After 10 weeks of TMSC treatment, mice were fasted for 8 h followed by injection of (**C**) glucose (2.0 g/kg) or (**D**) insulin (0.75 IU/kg) (*n* = 10). (**E**) Plasma insulin levels were measured after glucose (2.0 g/kg) injection (*n* = 10). (**F**) Secreted insulin levels were measured from isolated pancreatic islets after glucose treatment (*n* = 5). Data are means ± S.E.M. * *p* < 0.05, ** *p* < 0.01, *** *p* < 0.001.

As reported previously [17], TMSCs used in the present study were negative for CD34 and positive for CD73, CD90, and CD105 (Supplementary Figure S1A). In addition, TMSCs were efficiently differentiated into adipocytes, osteocytes, and chondrocytes under induction medium (Supplementary Figure S1B). To explore the therapeutic potential of TMSCs in T2DM, mice were fed a HFD for 6 weeks, and then TMSCs were injected intraperitoneally once every 2 weeks into HFD-induced T2DM mice while diets were maintained (Figure 1A). After 10 weeks of TMSC administration, body weight was significantly reduced (Figure 1B). Interestingly, TMSC administration improved HFD-induced glucose tolerance (Figure 1C) but did not affect insulin tolerance (Figure 1D). TMSC injection normalized HFD-induced increase in fasting plasma insulin levels and significantly increased glucose-stimulated plasma insulin levels (Figure 1E). Similarly, insulin secretion from isolated pancreatic islets in low glucose medium (2.2 mM glucose) was abnormally high in the HFD-fed group, and TMSC injection normalized insulin secretion function from isolated pancreatic islets (Figure 1F). 

### 3.2. TMSC Administration Preserved Pancreas Integrity Despite HFD

**Figure 2:**
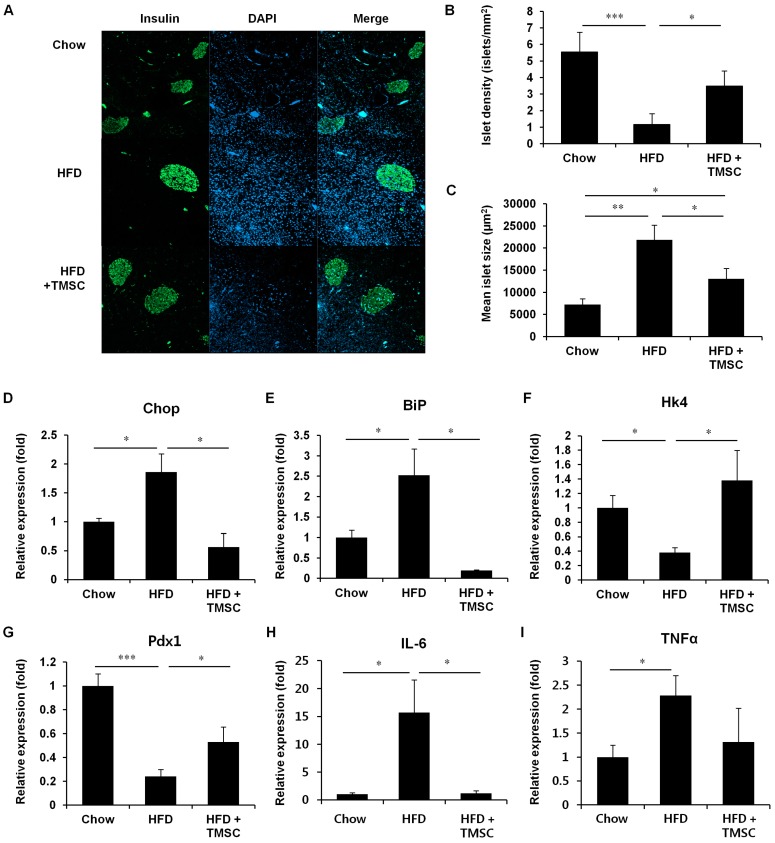
TMSC injection preserved pancreas integrity during HFD consumption. Mice were fed HFD for 6 weeks, and then TMSCs were injected intraperitoneally once every 2 weeks into HFD-induced diabetic mice while a normal or HFD diet was maintained. Mice were sacrificed after 10 weeks of TMSC administration, and pancreas tissues were extracted. (**A**) Thin (4 μm) pancreatic tissue sections were immunolabelled with a primary antibody for insulin followed by Cy2-conjugated secondary antibody incubation, and mounting with 4′,6-diamidino-2-phenylindole for nuclear staining. The image is a representative image of three independent experiments, 100× magnification. (**B**) Islet density and (**C**) size were calculated using ImageJ software (*n* = 5). Real-time polymerase chain reaction was performed to detect gene expression levels of (**D**) Chop, (**E**) BiP, (**F**) Hk4, (**G**) Pdx1, (**H**) IL-6, and (**I**) TNFα. BiP, binding immunoglobulin protein; Chop, C/EBP homologous protein; Hk4, hexokinase 4; IL-6, interleukin-6; Pdx1, pancreatic and duodenal homeobox 1, TNFα, tumor necrosis factor α. Values are mean ± S.E.M. (*n* = 5). * *p* < 0.05, ** *p* < 0.01, *** *p* < 0.001.

Because TMSC administration normalized pancreatic insulin secretion (Figure 1E,F), we further examined islet density and size in the pancreas (Figure 2A–C). HFD feeding decreased islet density but increased mean islet size compared with mice fed normal chow (Figure 2A–C). In accordance with insulin secretion data (Figure 1E,F), TMSC administration partially recovered both islet density and mean islet size (Figure 2A–C). Since ER stress is one of the main causes of β-cell dysfunction and death [1], we next examined levels of ER-stress markers, C/EBP homologous protein (Chop) and binding immunoglobulin protein (BiP) in isolated pancreatic islets (Figure 2D,E). TMSC injection alleviated the HFD-induced ER-stress response in pancreatic islets (Figure 2D,E). Because TMSC injection recovered the insulin secretion function in the pancreas (Figure 1E,F), real-time PCR was performed to measure the expression of genes related to glucose sensing and insulin secretion, such as hexokinase 4 (Hk4) and pancreatic and duodenal homeobox 1 (Pdx1) (Figure 2F,G). HFD feeding diminished gene levels of Hk4 and Pdx1, and this was partially recovered by TMSC injection (Figure 2F,G). Furthermore, we measured expression of inflammatory cytokines, including interleukin (IL)-6 and TNFα, in pancreas tissues (Figure 2H,I). TMSC injection reduced interleukin-6 (IL-6) mRNA expression significantly (Figure 2H). Expression of TNFα in TMSC injected group was also decreased compared with HFD group, but the difference was not significant (Figure 2I). These data suggest that TMSC injection alleviated the HFD-induced ER-stress and inflammation in pancreas, and improved the insulin secretion function of the pancreas. 

**Figure 3:**
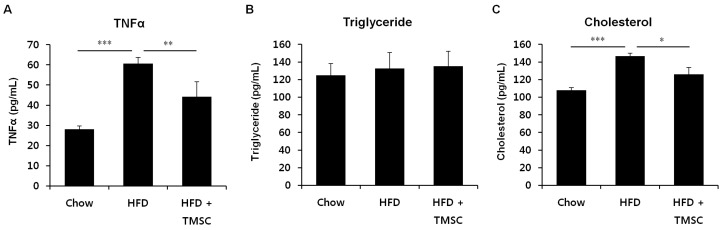
Serum TNFα and cholesterol levels were improved by TMSC administration. Mice were fed HFD for 6 weeks, and then TMSCs were injected intraperitoneally once every 2 weeks into HFD-induced diabetic mice while a normal or HFD diet was maintained. Mice were sacrificed after 10 weeks of TMSC administration and blood was obtained through heart puncture. (**A**) Serum TNFα levels were measured using an ELISA kit. Serum (**B**) triglyceride and (**C**) cholesterol levels were measured with a Reflotron Plus machine. Values are mean ± S.E.M. (*n* = 10). * *p* < 0.05, ** *p* < 0.01, *** *p* < 0.001.

**Figure 4:**
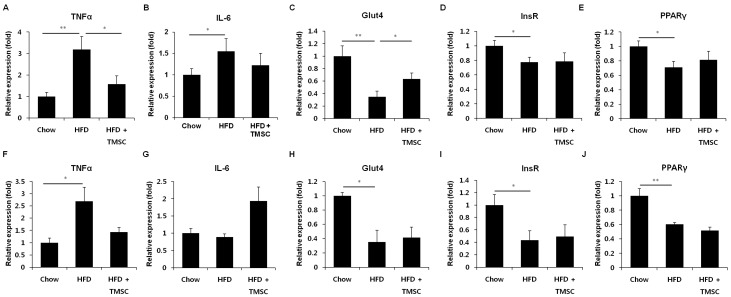
Hepatic and adipose changes in gene expressions associated with inflammation and insulin sensitivity. Mice were fed HFD for 6 weeks, and then TMSCs were injected intraperitoneally once every 2 weeks into HFD-induced diabetic mice while a normal or HFD diet was maintained. Mice were sacrificed after 10 weeks of TMSC administration and liver and adipose tissues were extracted. Real-time polymerase chain reaction was performed to detect gene expression levels of (**A**) TNFα, (**B**) IL-6, (**C**) Glut4, (**D**) InsR, and (**E**) PPARγ in liver. Gene expression levels of (**F**) TNFα, (**G**) IL-6, (**H**) Glut4, (**I**) InsR, and (**J**) PPARγ in adipose tissue were also analyzed similarly. Glut4, glucose transporter type 4; InsR, insulin receptor; IL-6, interleukin-6; PPARγ, Peroxisome proliferator-activated receptor gamma; TNFα, tumor necrosis factor α. Values are mean ± S.E.M. (*n* = 10). * *p* < 0.05, ** *p* < 0.01.

Next, we examined whether TMSC injection can alleviate HFD-induced metabolic alterations in whole-body (Figure 3). HFD feeding increased serum TNFα and cholesterol levels compared with chow diet controls, and TMSC injection partially reduced serum TNFα and cholesterol levels (Figure 3A). However, triglyceride levels were not significantly changed among three groups. These data indicate that TMSC injection improved whole-body inflammation and metabolism partially. Then, we investigated whether TMSC injection can also alleviate HFD-induced metabolic pathologies in peripheral tissues such as the liver and adipose tissue (Figure 4 and Supplementary Figure S2). While hepatic TNFα expression was significantly decreased upon TMSC injection (Figure 4A), hepatic IL-6 mRNA levels were not altered by TMSC injection (Figure 4B), suggesting only partial recovery of hepatic inflammation by TMSC injection. Hepatic Glut4 gene expression was partially recovered by TMSC injection (Figure 4C), but other hepatic gene expressions related with insulin sensitivity such as insulin receptor (InsR) and peroxisome proliferator-activated receptor gamma (PPARγ) were not significantly improved by TMSC injection (Figure 4C–E). In adipose tissue, gene expressions related with inflammation and insulin sensitivity were not improved by TMSC administration (Figure 4F–J). Then, we further analyzed hepatic gene expressions related with gluconeogenesis and glycogen degradation (Supplementary Figure S2A,B). While fructose-1,6-bisphosphatase 2 gene expression was partially normalized upon TMSC administration (Supplementary Figure S2A), the expression of most genes involved in gluconeogenesis (Supplementary Figure S2A) and glycogen degradation (Supplementary Figure S2B) were not significantly changed upon TMSC administration compared with the HFD group. In addition, lipid droplets displayed in hematoxylin and eosin-stained liver sections and lipocyte diameter in white adipose tissue were not altered upon TMSC injection (Supplementary Figure S2C,D). These data indicate that TMSC injection did not improve peripheral insulin resistance despite partial recovery of hepatic inflammation and are consistent with the insulin tolerance testing results (Figure 1D).

### 3.3. CM from TMSCs Also Improved HFD-Induced Glucose Tolerance

**Figure 5:**
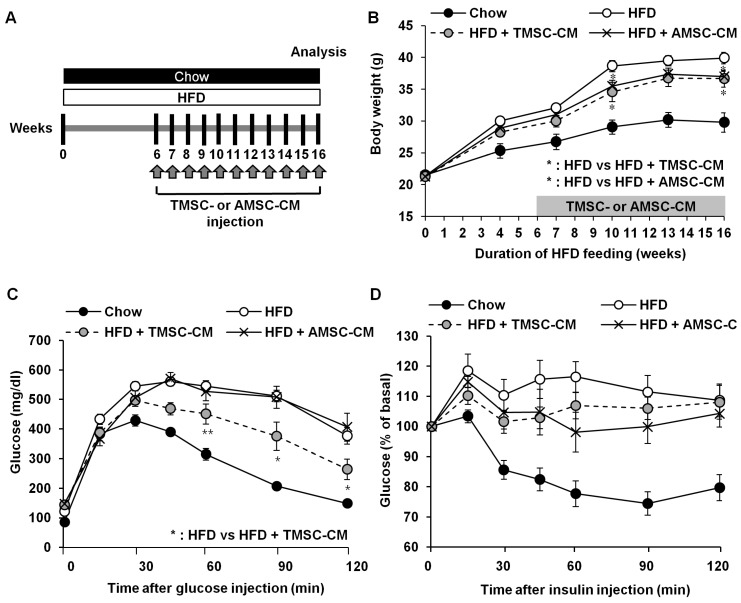
Conditioned medium derived from TMSCs (TMSC-CM) also improved HFD-induced glucose tolerance. (**A**) Mice were fed a HFD for 6 weeks, and then TMSC-CM was infused once weekly into HFD-induced diabetic mice while a normal or HFD diet was maintained. (**B**) Body weight was measured during normal chow or HFD feeding (*n* = 10). After 10 weeks of TMSC-CM infusion, mice were fasted for 8 h followed by injection of (**C**) glucose (2.0 g/kg) or (**D**) insulin (0.75 IU/kg) (*n* = 10). Data are mean ± S.E.M. * *p* < 0.05, ** *p* < 0.01.

Since the therapeutic efficacy of CM derived from TMSCs (TMSC-CM) was reported in previous studies [28,30] and PKH26 Red-stained TMSCs injected intraperitoneally were not observed in pancreatic slides (data not shown), the therapeutic potential of TMSC-CM was explored in our HFD-induced T2DM mouse model. We also compared the therapeutic efficacy of TMSC-CM with CM derived from AMSCs (AMSC-CM). MSC-CM was intravenously injected once a week into HFD-induced T2DM mice (Figure 5A). After 4 weeks of TMSC- or AMSC-CM administration, body weight started to be significantly reduced (Figure 5B). However, administration of only TMSC-CM, but not AMSC-CM, improved HFD-induced glucose intolerance after 10 weeks of CM injection (Figure 5C). Like TMSC injection (Figure 1D), infusion of TMSC-CM or AMSC-CM did not affect insulin tolerance (Figure 5D). Since only TMSC-CM administration alleviated HFD-induced glucose intolerance, we compared the effects of AMSC and TMSC on mRNA and protein levels using microarray and proteomic assays. Proteomic analysis detected 44 and 50 proteins in TMSC-CM (Supplementary Table S2) and AMSC-CM (Supplementary Table S3), respectively, and 21 proteins were detected in both TMSC- and TMSC-CM (Supplementary Tables S2 and S3). 23 proteins were only detected in TMSC-CM (Table 1). Among them (Table 1), only five genes corresponding to pro-matrix metalloproteinase-1, preprostromelysin, IGFBP5, laminin A3, and phospholipase A2 (PLA2) were detected in microarray data with ≥2.0-fold differences between TMSCs and AMSCs (Table 2). According to microarray data, 1040 genes were elevated with ≥2.0-fold differences in TMSCs compared with AMSCs (data not shown).

### 3.4. IGFBP5 was Critical for TMSC Protective Effects Against HFD-Induced Glucose Intolerance

**Figure 6:**
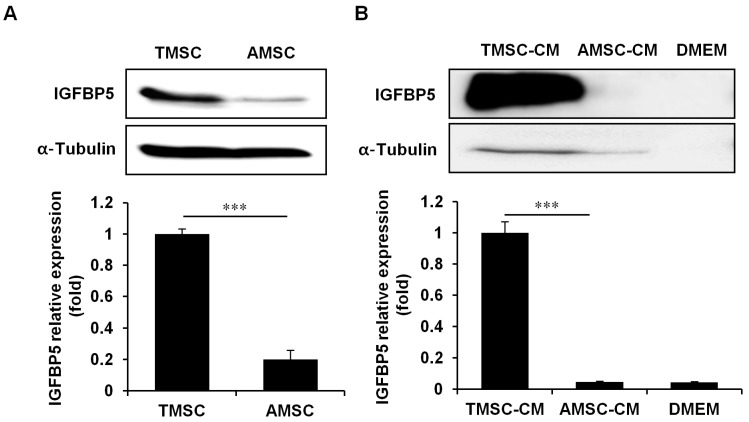
IGFBP5 protein expression and secretion were much higher in TMSCs compared with adipose tissue-derived mesenchymal stem cells (AMSCs). Western blot analyses of IGFBP5 in (**A**) cell lysates and (**B**) 20-fold concentrated CM. The image is a representative image of three independent experiments. Bands were quantified using ImageQuant software. Values are mean ± S.E.M. (*n* = 4). *** *p* < 0.001.

**Figure 7:**
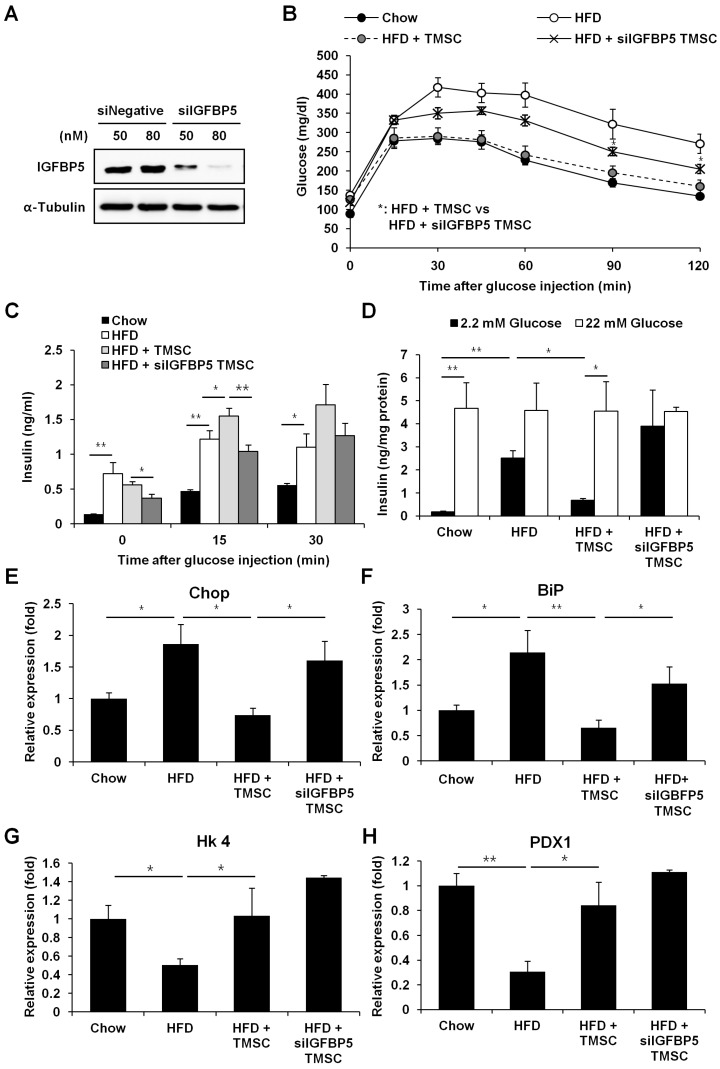
IGFBP5 expression was critical for the protective effects of TMSCs in HFD-induced glucose intolerance. The efficacy of IGFBP5-knockdown TMSCs in type 2 diabetes mellitus (T2DM) was investigated using siRNA. (**A**) Western blot analyses of IGFBP5 in TMSCs after siRNA treatment. The image is a representative image of three independent experiments. Mice were fed a HFD for 6 weeks, and then control TMSCs or IGFBP5-knockdown TMSCs were injected intraperitoneally once every 2 weeks into HFD-induced diabetic mice while a normal or HFD diet was maintained. After 10 weeks of TMSC administration, mice were fasted for 8 h followed by injection of (**B**) glucose (2.0 g/kg) (*n* = 10). (**C**) Plasma insulin levels were measured after glucose (2.0 g/kg) injection (*n* = 10). (**D**) Secreted insulin levels were measured from isolated pancreatic islets after glucose treatment (*n* = 5). Real-time polymerase chain reaction was performed to detect gene expressions of (**E**) Chop, (**F**) BiP, (**G**) Hk4, and (h) Pdx1. BiP, binding immunoglobulin protein; Chop, C/EBP homologous protein; Hk4, hexokinase 4; Pdx1, pancreatic and duodenal homeobox 1. Values are mean ± S.E.M. (*n* = 5). * *p* < 0.05, ** *p* < 0.01.

We decided to focus on IGFBP5, based on its association with insulin-like growth factor (IGF)-1 [31] and its positive effects on MSC function [32,33]. Indeed, IGFBP5 protein expression and secretion were highly elevated in TMSCs compared with AMSCs (Figure 6A,B). The efficacy of IGFBP5-knockdown TMSCs in T2DM was investigated using siRNA. *IGFBP5* gene expression was successfully downregulated in TMSCs following 80 nM siRNA treatment (Figure 7A), and IGFBP5 knockdown partially reversed the protective effects of TMSCs on HFD-induced glucose intolerance (Figure 7B). Compared with the control TMSC administration group, IGFBP5-knockdown TMSCs decreased plasma insulin levels (Figure 7C) and abrogated TMSC injection-induced normalization of insulin secretion from isolated pancreatic islets (Figure 7D). IGFBP5 knockdown in TMSCs also reversed TMSC injection-induced normalization of Chop and BiP expression during HFD feeding (Figure 7E,F). However, IGFBP5 knockdown in TMSCs did not affect TMSC injection-induced normalization of Hk4 and Pdx1 expression during HFD feeding (Figure 7G,H). These data imply that IGFBP5 expression is important for TMSCs to modulate HFD-induced ER stress and exert therapeutic effects on HFD-induced glucose intolerance.

### 3.5. IGFBP5 Knockdown in TMSCs Diminished Cell Proliferation

Considering an important role of IGFBP5 in MSCs [32,33], the effects of IGFBP5 knockdown in TMSCs were evaluated. IGFBP5 knockdown in TMSCs suppressed TMSC proliferation before and after palmitate treatment (Supplementary Figure S3A,B) but had no effect on expressed levels of MSC markers such as CD73 and CD90, or the lack of CD34 expression (Supplementary Figure S3C).

## 4. Discussion

Obesity is among the most common comorbidities and causes of T2DM. Feeding animals a diet high in fat is a model that mimics the eating habits leading to obesity in humans, thus providing insight into the mechanisms of T2DM. Common pathological features include weight gain, fasting and/or postprandial hyperglycaemia, a shift from initial hyperinsulinaemia to hypoinsulinaemia due to pancreatic β-cell exhaustion, insulin resistance, changes in lipid metabolism-regulating hormones, increased inflammation in associated organs, and lipid droplet accumulation or fibrosis in related tissues [25]. In the present study, HFD-fed mice were used to investigate the therapeutic potential of TMSCs in T2DM. TMSC injection successfully normalized HFD-induced glucose intolerance and pancreatic insulin secretion. Several studies already reported the therapeutic effects of MSCs in T2DM [6,23]. BM-MSC transplantation ameliorated hyperglycaemia in diabetic mice by promoting β-cell regeneration, and MSC-CM treatment promoted islet cell proliferation [34]. AMSC infusion also improved HFD-induced glucose intolerance and preserved β-cell mass in a HFD-induced T2DM mouse model [23]. Therefore, the cytoprotective and repairing effects of TMSCs on pancreas are similar to those of other MSCs. The unexpected result that TMSC injection did not improve peripheral insulin resistance, and adiposity in liver and adipose tissues was not consistent with previous studies with other MSCs [22,23]. Infusion of BM-MSC restored GLUT4 protein levels in peripheral tissues including liver and adipose tissue and led improvement of peripheral insulin sensitivity [22]; however, TMSC injection partially recovered Glut4 expression only in liver, and not in adipose tissue. AMSC infusion recovered hepatic InsR and PPARγ gene expressions [23], but TMSC injection did not restore InsR and PPARγ levels in both liver and adipose tissue. Similarly, TMSC injection did not reduce HFD-induced lipid accumulation in peripheral tissues, while AMSC infusion reduced fat liver and adipositiy [23]. This might be attributable to either differences in MSC origin or variable MSC administration methods [22,23]. The present study is the first investigation of the possibility of intraperitoneal human MSC therapy for T2DM. Considering the possible fatal complication of intravenous cell infusion, intraperitoneal injection may provide an alternative method for cell administration in diabetes, as investigated in lung injury [35] and colitis [36]. 

Obesity-induced chronic inflammation is a key component in the pathogenesis of insulin resistance [37]. TMSC injection significantly reduced serum and hepatic TNFα levels, but did not affect IL-6 level. These data indicate partial relief of inflammation by TMSC treatment, which could also affect glucose clearance. Since AMSC infusion significantly decreased hepatic IL-6 expression [23], degree of inflammation relief might be different depending on MSC sources. Anti-inflammatory and immune-modulatory effects of TMSCs have been well reported previously [8,38,39]. TMSCs suppress T-cell activation and cytokine secretion [8], and inhibit the differentiation, maturation, and function of dendritic cells [38]. Despite the partial relief of inflammation, TMSC injection did not improve peripheral insulin sensitivity. This is in accordance with the previous study; TNF-α blockade with etanercept in T2DM patients had a significant beneficial effect on systemic inflammatory markers, but no improvement of metabolic insulin sensitivity was observed [40]. Therefore, partial restoration of inflammatory response by TMSCs would not be sufficient to improve peripheral insulin sensitivity.

In T2DM, both chronic hyperglycaemia and hyperlipidaemia disrupt ER homeostasis and induce an abnormal unfolded protein response and β-cell death [1]. TMSC administration alleviated HFD-induced ER stress in pancreatic islets, and IGFBP5 knockdown in TMSCs diminished the protective effect of TMSCs against ER stress of pancreatic islets. IGFBP5 exerts biological actions both dependent on and independent of IGF-I [41]. IGF-I can positively influence β-cell growth and development, and the IGFBP family has been postulated to modulate IGF activity by various mechanisms including protecting IGFs from proteolytic degradation, targeting serum IGFs to specific tissues, and regulating local IGF availability to receptors by sequestration in extracellular storage pools [41,42]. Besides important roles of IGFBP5 in cell survival and proliferation [43,44], it may also help regulate glucose homeostasis because Igfbp5-deficient mice exhibit impaired glucose tolerance [41]. IGFBP5 knockdown in TMSCs abrogated the protective effect of TMSCs in HFD-induced ER stress of pancreatic islets, suggesting that IGFBP5 may regulate glucose homeostasis via regulating the ER-stress response in pancreatic islets. There is no clearly described functional role of IGFBP5 in ER stress. According to a previous study, among IGFBPs, only IGFBP3 and IGFBP5 bind GRP78/BiP, a master regulator of the unfolded protein response, and IGFBP3-expressing cells exhibit enhanced growth and survival when treated with two pharmacological inducers of ER stress (tunicamycin and 2-deoxyglucose) [45]. Whether IGFBP5 can modulate ER stress via binding to BiP and whether IGFBP5 infusion can improve T2DM symptoms require further investigation.

In the present study, only TMSC-CM, and not AMSC-CM, administration alleviated HFD-induced glucose intolerance. Several studies previously reported therapeutic effects of AMSCs in animal models of diabetes. Relative protein expression levels of insulin receptor substrate-1 and GLUT4 were augmented in the canine AMSC-CM-treated group compared to insulin resistant models, and fibroblast growth factor-1 from canine AMSC-CM has been suggested as an alternative insulin sensitizer [46]. AMSC infusion also reduced diabetic renal injury in diabetic rats with no improvement in endocrine pancreas function via glial cell line-derived neurotrophic factor secretion [47]. In the present study, proteomic analysis detected 50 proteins in AMSC-CM, but fibroblast growth factor-1 and glial cell line-derived neurotrophic factor were not detected. Donor variation might attribute to the discrepancy.

Similar with AMSCs, TMSCs were negative for CD34 and positive for CD73, CD90, and CD105. In addition, TMSCs can be successfully differentiated into adipocytes, osteocytes, and chondrocytes similar with other MSCs. In the previous study [14,15], we also reported similar differentiating capacity of AMSCs and TMSCs into insulin-secreting cells [14], and surface marker screening revealed several common markers expressed in both AMSCs and TMSCs [15]. In the present study, proteomic analysis also identified 21 proteins detected in both TMSC- and TMSC-CM. However, RNA sequencing and surface marker screening also identified difference between AMSCs and TMSCs previously [15]. Proteomic analysis identified 27 proteins and 21 proteins, which are detected only in AMSC- and TMSC-CM, respectively. Especially, IGFBP5 gene expression and its protein secretion were much higher in TMSCs compared with AMSCs. An important role of IGFBP5 in MSCs was reported previously [32,33]. IGFBP5 enhanced the osteogenic differentiation potentials of periodontal ligament stem cells and Wharton’s jelly of umbilical cord stem cells via the Jun N-terminal kinase and extracellular regulated protein kinase signalling pathways [32]. In addition, local application of recombinant IGFBP5 protein enhanced the migration, chemotaxis, osteo-/dentinogenic differentiation, and cell proliferation of MSCs under inflammatory conditions [33]. Similarly, IGFBP5 knockdown in TMSCs suppressed TMSC proliferation. Therefore, we cannot exclude the possibility of diminished TMSC survival and proliferation upon intraperitoneal injection, when IGFBP5 is downregulated in TMSCs. 

We previously demonstrated the differentiating potential of TMSCs into insulin-secreting cells [14]. This requires a specific medium containing insulin, transferrin, selenium, nicotinamide, glucagon-like peptide, and exendin-4. Furthermore, insulin-producing cells from TMSCs are de-differentiated into non-insulin-producing cells after withdrawal of differentiation factors [14]. Therefore, differentiation of TMSCs into insulin-secreting cells is not likely to occur in intraperitoneally injected MSCs in vivo. Bazhanov et al. reported that intraperitoneally injected MSCs are rapidly surrounded by host immune cells to form aggregates [48]. Indeed, we found that the injected TMSCs were rarely recruited to the pancreas by tracking injected fluorescence-labelled TMSCs (data not shown). Instead, it is much more plausible that indirect and paracrine functions of TMSCs were responsible for the observed improvements in HFD-induced glucose intolerance and insulin secretion. In the present study, TMSC-CM also had a similar capacity and properties to regulate glucose in HFD-induced T2DM mice, suggesting a major paracrine contribution. Several paracrine factors from MSCs have been identified, including vascular endothelial growth factor alpha, platelet-derived growth factor, angiopoietin-1, and insulin-like growth factor [6,49]. We also previously reported several paracrine factors secreted from TMSCs [28,30,39]. For instance, osteoprotegerin secreted from TMSCs effectively inhibited interactions between T-helper type 17 cells and osteoclasts [30]. Interferon-β secreted from TMSCs increased programmed death-ligand 1 expression on T-cells [39]. TMSC-CM also inhibited the skeletal muscle cell-derived pro-fibrogenic effect via production of the interleukin-1 receptor antagonist, which inhibits interleukin-1 signalling [28]. In the present study, 44 and 50 proteins were newly identified in TMSC- and AMSC-CM, respectively, and these data will provide a fundamental basis for paracrine analysis of TMSCs and AMSCs. 

## 5. Conclusions

In summary, the present study demonstrated that TMSC administration improved HFD-induced glucose intolerance by enhancing insulin secretion. IGFBP5 secretion after TMSC injection played an important role in enhanced insulin secretion and alleviated ER stress. These results confirm the critical paracrine effects of TMSCs and provide new insights into the administration of TMSCs or TMSC-CM as a novel T2DM therapy. 

## Figures and Tables

**Table 1:** Proteins detected only in TMSC-CM, but not in AMSC-CM.

Protein Name
14-3-3 protein epsilon isoform
71 Kd heat shock cognate protein
Beta-2 microglobulin
C1 esterase
Calcium binding protein Cab45 precursor
Cathepsin D
Decorin
Fibulin-1D
Insulin-like growth factor-binding protein 4
Insulin-like growth factor-binding protein 5
Insulin-like growth factor-binding protein 7
Laminin A3
Laminin B2
Nucleobindin
Phospholipase A2
Phospholipid transfer protein
Preprostromelysin
Pro-matrix metalloproteinase-1
Quiescent cell proline dipeptidase
Quiescin
Ras GTPase-activating-like protein
Transketolase
Triosephosphate isomerase

**Table 2:** Microarray data of genes corresponding to proteins detected only in TMSC-CM, but not in conditioned medium derived from adipose tissue-derived mesenchymal stem cells (AMSC-CM).

Gene Number	Gene Symbol	Gene Description	TMSC/AMSC (fold)
NM_001145938	*MMP1*	Matrix metalloproteinase-1	70.7
NM_002422	*MMP3*	Preprostromelysin	6.27
NM_000599	*IGFBP5*	Insulin-like growth factor-binding protein 5	2.18
NM_000227	*LAMA3*	Laminin A3	2.09
NM_001311193	*PLA2G4A*	Phospholipase A2	2.07
NM_001996	*FBLN1*	Fibulin 1	1.94
NM_001552	*IGFBP4*	Insulin-like growth factor-binding protein 4	1.46
NM_001004128	*QSOX1*	Quiescin sulfhydryl oxidase 1	1.29
NM_005013	*NUCB2*	Nucleobindin 2	1.03
NM_001242920	*PLTP*	Phospholipid transfer protein	−1.03
NM_002292	*LAMB2*	Laminin B2	−1.40
NM_001734	*C1S*	C1 esterase	−1.66
NM_001253835	*IGFBP7*	Insulin-like growth factor-binding protein 7	−1.75
NM_001920	*DCN*	Decorin	−2.27
NM_006761	*YWHAE*	Tyrosine 3-monooxygenase/tryptophan 5-monooxygenase activation protein epsilon	ND ^1^
NM_006597	*HSPA8*	Heat shock protein family A	ND
NM_004048	*B2M*	Beta-2 microglobulin	ND
NM_016176	*SDF4*	Stromal cell derived factor 4	ND
NM_001909	*CTSD*	Cathepsin D	ND
NM_013379	*DPP7*	Dipeptidyl peptidase 7	ND
NM_003870	*IQGAP1*	IQ motif containing GTPase activating protein 1	ND
NM_001064	*TKT*	Transketolase	ND
NM_001258026	*TPI1*	Triosephosphate isomerase 1	ND

^1^ ND: Not detected in microarray data.

## Supplementary Materials

The following are available online at https://www.mdpi.com/2073-4409/8/4/368/s1, Figure S1: Characterization of TMSCs; Figure S2: Obesity-associated metabolic pathologies in peripheral tissues were not relieved by TMSCs; Figure S3: Characterization of IGFBP5-knockdown TMSCs; Table S1: Primers used in real-time PCR; Table S2: Proteins detected in TMSC-CM; Table S3: Proteins detected in AMSC-CM.

## Abbreviations

AMSC	adipose tissue-derived MSC
AMSC-CM	conditioned medium derived from AMSCs
BiP	binding immunoglobulin protein
BM-MSC	bone marrow-derived MSC
cDNA	complementary DNA
CHOP	C/EBP homologous protein
CM	conditioned medium
ER	endoplasmic reticulum
Glut4	glucose transporter type 4
HFD	high fat diet
Hk4	hexokinase 4
HKRB	HEPES-added Krebs-Ringer bicarbonate buffer
IGF	insulin-like growth factor
IGFBP	insulin-like growth factor-binding protein
InsR	insulin receptor
IL-6	interleukin-6
MS	mass spectrometry
MSC	mesenchymal stem cell
PCR	polymerase chain reaction
Pdx1	pancreatic and duodenal homeobox 1
PLA2	phospholipase A2
PPARγ	Peroxisome proliferator-activated receptor gamma
STZ	streptozotocin
T2DM	type 2 diabetes mellitus
TMSC	tonsil-derived mesenchymal stem cell
TMSC-CM	conditioned medium derived from TMSCs
TNFα	tumor necrosis factor α
